# Joint association of postoperative visual acuity status and baseline loneliness with cognitive impairment after cataract surgery in older adults: a retrospective cohort study

**DOI:** 10.3389/fmed.2026.1948093

**Published:** 2026-09-15

**Authors:** Xiaoman Liu, Haixia Ji, Xiaohong Jin

**Affiliations:** Department of Ophthalmology, Affiliated Hospital of Nantong University, Nantong, China

**Keywords:** age-related cataract, cataract surgery, cognitive impairment, loneliness, visual recovery

## Abstract

**Background:**

Evidence on the joint relationship of postoperative visual acuity and loneliness with cognition after cataract surgery remains limited. This study examined whether postoperative visual acuity status and loneliness were jointly associated with cognitive impairment in older patients with age-related cataract.

**Methods:**

This single-center retrospective cohort study included patients aged 65 years or older who underwent cataract surgery. Four groups were defined by postoperative best-corrected visual acuity (BCVA) status and baseline loneliness measured with the University of California, Los Angeles (UCLA) Loneliness Scale. Cognitive impairment at 6 months was assessed using the education-adjusted Montreal Cognitive Assessment (MoCA). Multivariable logistic regression, interaction analyses, and Firth penalized logistic regression were used.

**Results:**

Among 377 patients, 79 (21.0%) had cognitive impairment. The proportions across the four groups were 4.9%, 18.3%, 7.4%, and 69.7%. Using good postoperative BCVA without loneliness as the reference, adjusted odds ratios were 5.39 [95% confidence interval (CI), 1.91–16.22] for good BCVA with loneliness, 1.85 (95% CI, 0.48–6.60) for poor BCVA without loneliness, and 88.50 (95% CI, 27.43–338.63) for poor BCVA with loneliness. Positive interaction was observed on multiplicative and additive scales. Firth regression reduced the estimate for the dual-exposure group to 62.18 (95% CI, 20.67–218.82) without altering the overall pattern. Higher UCLA scores were associated with greater MoCA decline (r = −0.503, *P* < 0.001).

**Conclusion:**

Baseline loneliness was associated with higher odds of cognitive impairment, particularly among patients with poor postoperative BCVA. The large effect estimates and exploratory interaction findings should be interpreted cautiously and require confirmation in larger prospective cohorts.

## 1 Introduction

Age-related cataract (ARC) remains one of the most common causes of visual impairment among older adults and represents an increasing public health burden as populations age (1, 2). Cataract surgery is an effective treatment for restoring vision, and improvements in best-corrected visual acuity (BCVA) are associated with better visual function and quality of life (3, 4). In older patients, however, postoperative outcomes extend beyond vision alone. Cognitive function, ability to perform daily activities, emotional status, and social engagement may all influence how patients adapt after surgery. Cognitive impairment is particularly relevant in this population because it may affect independence, adherence to follow-up, and the broader course of postoperative recovery.

A growing body of evidence points to a close relationship between visual function and cognition. In older adults, visual impairment has been associated with poorer cognitive performance and a faster decline across several cognitive domains (5, 6). Cataract-related vision loss may reduce sensory input, limit mobility, and make reading or other visually demanding activities more difficult. It may also restrict social participation. Several clinical studies have reported stable or improved cognitive performance after cataract surgery, although the findings have not been entirely consistent across populations or cognitive assessment methods (7–9). Most previous studies have focused on whether patients underwent cataract surgery, rather than on the level of vision achieved afterward. This distinction matters because patients receiving the same procedure may have substantially different postoperative best-corrected visual acuity, which may in turn be relevant when assessing cognitive outcomes after surgery.

Loneliness may also contribute to cognitive vulnerability in later life. Although loneliness is a subjective feeling and social isolation refers to an objective lack of social contact, the two are closely related. Longitudinal and epidemiological studies have linked loneliness to poorer cognitive performance, cognitive decline, and dementia-related outcomes (10–12). Similar findings have been reported in Chinese older adults, in whom loneliness has been negatively associated with cognitive function and may partly mediate the relationship between social engagement and cognition (13). This issue may be particularly relevant after cataract surgery. Improved vision does not necessarily lead to greater social participation or cognitive stimulation, especially when loneliness persists. Conversely, poor postoperative visual acuity may continue to limit mobility, confidence, and interaction with the surrounding environment.

Few studies have examined postoperative visual acuity and loneliness together in relation to cognitive outcomes after cataract surgery. Considering both factors may provide a more complete picture of postoperative cognitive vulnerability than assessing either one alone. We therefore investigated the joint association of postoperative visual acuity and baseline loneliness with cognitive impairment at 6 months in older patients with ARC. Patients were classified into four joint exposure groups according to postoperative visual acuity status and loneliness, and changes in Montreal Cognitive Assessment (MoCA) scores were also examined. This approach may help identify patients who cloud benefit from closer cognitive and functional follow-up after surgery.

## 2 Methods

### 2.1 Study design and participants

This single-center retrospective cohort study was conducted at the Affiliated Hospital of Nantong University. Potentially eligible cataract surgery records were identified through the electronic medical record system. A total of 502 records were screened.

Patients were eligible if they were 65 years of age or older, had a clinical diagnosis of age-related cataract, underwent cataract surgery at the study center, and had available records of preoperative and postoperative visual assessment, preoperative loneliness assessment, baseline cognitive assessment, and cognitive follow-up at 6 months after surgery. Patients were excluded if they had non-age-related or secondary cataract, a previous diagnosis of dementia or mild cognitive impairment, cognitive impairment at baseline based on the education-adjusted MoCA cutoff, severe neurological disease, severe psychiatric illness, or an inability to complete the required assessments. Patients with ocular disease expected to substantially limit postoperative best-corrected visual acuity independently of cataract were also excluded. These conditions included advanced age-related macular degeneration, advanced glaucoma with optic nerve or visual field damage, visually significant corneal opacity, severe diabetic retinopathy or diabetic macular edema, retinal detachment, and optic neuropathy. Mild or clinically stable ocular conditions were retained when they were not considered likely to prevent reliable postoperative visual assessment. These included early age-related macular degeneration, mild non-proliferative diabetic retinopathy without macular edema, medically controlled glaucoma without advanced optic nerve damage, and mild corneal disease. Retained conditions were recorded as ocular comorbidities. Intraoperative and postoperative complications were not used as preoperative exclusion criteria and were recorded separately. The patient was the unit of analysis, and only one index eye was included for each participant. For patients who underwent sequential bilateral cataract surgery during the study period, the first-operated eye was selected as the index eye. The fellow-eye record was excluded and was not treated as an independent observation. Patients were also excluded if key baseline variables, postoperative best-corrected visual acuity, or the 6-month cognitive assessment were unavailable. The study protocol was approved by the Ethics Committee of the Affiliated Hospital of Nantong University (approval no. 2025-K141-01). The study was conducted in accordance with the Declaration of Helsinki and reported following the Strengthening the Reporting of Observational Studies in Epidemiology (STROBE) guidelines (14).

### 2.2 Data collection and clinical variables

Data were retrospectively extracted from the electronic medical record system, ophthalmic examination records, operative notes, and follow-up assessment forms using a structured data collection sheet. Baseline information was obtained from records completed before cataract surgery and included age, sex, education duration, systemic comorbidities, ocular comorbidity, cognitive function, loneliness, and functional status. The systemic comorbidities considered in the analysis were hypertension, diabetes mellitus, history of stroke, depression, and hearing impairment.

All included index eyes underwent phacoemulsification with intraocular lens implantation. Information on the surgical procedure and any intraoperative complications was obtained from the operative records. Pre-existing ocular comorbidities were identified from diagnoses documented before surgery, whereas intraoperative and postoperative complications were extracted separately from the operative notes and postoperative follow-up records. For the statistical analyses, ocular comorbidity and postoperative complication were each recorded as present or absent. BCVA was obtained from the final preoperative ophthalmic examination and the routine 1-month postoperative assessment. When more than one postoperative BCVA measurement was available, the value recorded closest to 30 days after surgery was used. Decimal visual acuity values were converted to the logarithm of the minimum angle of resolution (LogMAR). Postoperative visual acuity status was determined from the postoperative LogMAR value. Visual gain was calculated as preoperative LogMAR minus postoperative LogMAR, with a larger positive value indicating greater improvement in visual acuity.

Loneliness was assessed at the preoperative baseline visit using the University of California, Los Angeles Loneliness Scale (UCLA Loneliness Scale). Cognitive function was evaluated with the MoCA at the same preoperative visit and again at approximately 6 months after surgery. Both assessments were administered by trained ophthalmic nurses using standardized instructions and consistent scoring procedures. Baseline functional status was assessed using the Barthel Index. Change in cognitive function was calculated as the 6-month MoCA score minus the baseline score; negative values indicated cognitive decline, and more negative values represented a greater decline over follow-up.

### 2.3 Definition of postoperative visual acuity status, baseline loneliness, and joint exposure groups

Postoperative visual status was defined using the best-corrected visual acuity of the index eye recorded at the routine 1-month postoperative assessment and expressed as the LogMAR. For patients with more than one postoperative visual acuity record, the measurement closest to 30 days after surgery was used. The index eye was the first-operated eye in patients who underwent sequential bilateral cataract surgery. Decimal visual acuity values were converted using the formula LogMAR = −log10(decimal visual acuity). Postoperative visual status was categorized using a literature-based operational cutoff of 0.3 LogMAR, equivalent to approximately 20/40 Snellen acuity or a decimal visual acuity of 0.5. This threshold has been used in previous cataract outcome studies to distinguish good from poor postoperative best-corrected visual acuity. A postoperative LogMAR value of 0.3 or lower was classified as good postoperative visual status, whereas a value greater than 0.3 was classified as poor postoperative visual status (15).

Baseline loneliness was assessed at the preoperative visit using the 20-item UCLA Loneliness Scale, Version 3. Each item was rated on a four-point scale, with positively worded items reverse-scored according to the standard scoring procedure. Total scores ranged from 20 to 80, with higher scores indicating greater loneliness (16). Because no universally accepted diagnostic cutoff has been established for this scale, a literature-based operational threshold of 44 or higher was used to define baseline loneliness, consistent with previous studies in older adult populations (17, 18). Postoperative visual status and baseline loneliness were combined to form four joint exposure groups: G1, good postoperative visual status without baseline loneliness; G2, good postoperative visual status with baseline loneliness; G3, poor postoperative visual status without baseline loneliness; and G4, poor postoperative visual status with baseline loneliness. G1 represented patients without either exposure and was used as the reference group in the regression analyses.

### 2.4 Outcome assessment

The primary outcome was cognitive impairment at the 6-month postoperative follow-up. Cognitive function was assessed before cataract surgery and again 6 months after surgery using the same validated Chinese-language version of the 30-point MoCA. Scores ranged from 0 to 30, with higher scores indicating better cognitive function. Following the standard scoring procedure, one point was added for participants with 12 years of education or less, without allowing the adjusted score to exceed 30. The same education correction was applied at both assessment time points. Cognitive impairment at follow-up was operationally defined as an education-adjusted MoCA score below 26 (19–21). Change in cognitive function was examined as a continuous secondary outcome. It was calculated by subtracting the education-adjusted baseline MoCA score from the education-adjusted 6-month score. Negative values indicated cognitive decline, and more negative values reflected a greater decline over follow-up. This change score was used to assess its association with baseline loneliness severity and to compare cognitive change across the four joint exposure groups.

### 2.5 Statistical analysis

Statistical analyses were performed using R software, version 4.5.2. Continuous variables are presented as mean ± standard deviation, and categorical variables as counts and percentages. Differences across the four joint exposure groups were examined using Welch's one-way analysis of variance for continuous variables and Fisher's exact test for categorical variables. Comparisons between patients with and without cognitive impairment were made using Welch's *t* test and Fisher's exact test, as appropriate.

The proportion of patients with cognitive impairment was calculated for each joint exposure group, with 95% confidence intervals estimated using the Wilson method. Binary logistic regression was used to examine the association between the joint exposure groups and 6-month cognitive impairment. Patients with good postoperative visual acuity status and no baseline loneliness (G1) served as the reference group. Odds ratios (ORs) and 95% confidence intervals (CIs) were reported. The crude model included no covariates. Model 1 adjusted for age, sex, and education duration, and Model 2 additionally adjusted for baseline MoCA score. The fully adjusted model further included the Barthel Index, ocular comorbidity, postoperative complication, hypertension, diabetes mellitus, history of stroke, depression, and hearing impairment. Because the study focused on the joint association of postoperative visual acuity status and baseline loneliness, interaction was also assessed formally. Multiplicative interaction was evaluated by adding a product term between poor postoperative visual acuity status and baseline loneliness to the fully adjusted logistic regression model. The interaction was expressed as a ratio of odds ratios, and statistical significance was assessed by a 1-degree-of-freedom likelihood ratio test comparing models with and without the interaction term. Additive interaction was examined on the risk-ratio scale using modified Poisson regression with robust standard errors. Relative excess risk due to interaction (RERI), attributable proportion due to interaction (AP), and the synergy index (SI) were calculated, with 95% CIs obtained using the delta method. Values of 0 for RERI and AP and 1 for SI indicate no additive interaction.

Several sensitivity analyses were undertaken to assess the stability of the main findings. Alternative covariate-adjustment models were fitted using the same joint exposure groups. Model A corresponded to the fully adjusted model described above. Model B adjusted for age, sex, education duration, baseline MoCA score, Barthel Index, history of stroke, and depression, whereas Model C adjusted for age, sex, education duration, and baseline MoCA score. Given the small number of outcome events in some exposure groups and the large OR observed for the dual-exposure group, Firth penalized logistic regression was additionally performed using the same covariates as the fully adjusted model. Firth estimates, 95% CIs, and *P* values were obtained from profile penalized likelihood.

Cognitive change was also examined as a continuous outcome. Change in MoCA score was defined as the education-adjusted 6-month score minus the education-adjusted baseline score, with more negative values indicating greater cognitive decline. Its association with the UCLA Loneliness Scale score was assessed using Pearson correlation, and fitted linear trends were displayed according to postoperative visual acuity status. Patients with missing key baseline, postoperative visual acuity, cognitive outcome, or required covariate data were excluded according to the eligibility and data-completeness criteria described above. All statistical tests were two-sided, and *P* < 0.05 was considered statistically significant.

## 3 Results

### 3.1 Patient selection and study cohort

A total of 502 potentially eligible cataract surgery records were identified from the hospital electronic medical record system. After eligibility review, 87 records were excluded because they did not meet the inclusion criteria, met one or more exclusion criteria, or represented duplicate or fellow-eye records from patients who underwent bilateral cataract surgery. This left 415 unique patients for baseline data review. Among these patients, 22 were excluded because preoperative visual, loneliness, cognitive, clinical, or covariate data were incomplete. Of the 393 patients with complete baseline data, a further 16 were excluded because postoperative best-corrected visual acuity or the 6-month Montreal Cognitive Assessment was unavailable, including patients who were lost to follow-up. The final analytic cohort comprised 377 patients (Figure 1).

**Figure 1:**
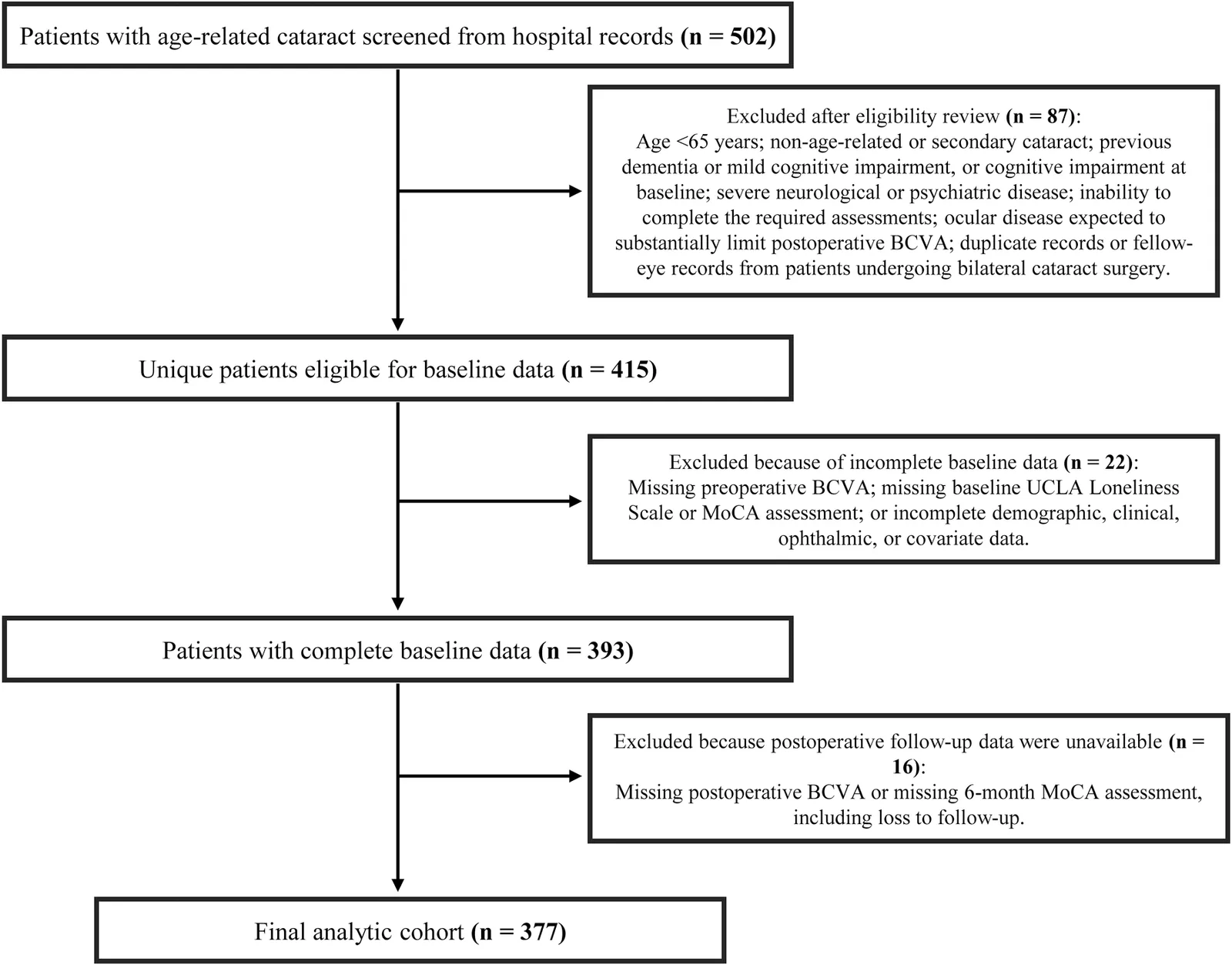
Flowchart of patient selection.

### 3.2 Clinical characteristics according to joint exposure groups

The final cohort comprised 377 patients: 162 in G1, 71 in G2, 68 in G3, and 76 in G4. The mean age was 77.0 ± 7.1 years, and 234 patients (62.1%) were women. Age, sex, education duration, and baseline Montreal Cognitive Assessment score did not differ significantly across the four groups. Postoperative BCVA and UCLA Loneliness Scale score, which were used to define the joint exposure groups, differed across groups by design. Preoperative best-corrected visual acuity and visual acuity gain also varied significantly (all *P* < 0.001). G4 had the highest postoperative LogMAR value and the lowest Barthel Index, whereas G1 had a lower postoperative LogMAR value and a higher Barthel Index. The distributions of ocular comorbidity, diabetes mellitus, depression, and hearing impairment also differed among the groups. No significant between-group differences were observed for postoperative complications, hypertension, or history of stroke (Table 1).

**Table 1:** Clinical characteristics of patients according to joint exposure groups.

	Joint exposure group					
Variable	Overall *N* = 377	G1 *N* = 162	G2 *N* = 71	G3 *N* = 68	G4 *N* = 76	*P* value
Age, years	77.0 ± 7.1	77.2 ± 7.0	76.7 ± 7.4	75.9 ± 7.2	77.9 ± 7.2	0.403
Sex						0.735
Male	143 (37.9%)	65 (40.1%)	26 (36.6%)	27 (39.7%)	25 (32.9%)	
Female	234 (62.1%)	97 (59.9%)	45 (63.4%)	41 (60.3%)	51 (67.1%)	
Education duration, years	8.5 ± 3.9	8.5 ± 3.8	8.6 ± 4.1	8.8 ± 4.2	8.3 ± 3.9	0.923
Preoperative BCVA, LogMAR	0.68 ± 0.18	0.62 ± 0.19	0.67 ± 0.17	0.72 ± 0.15	0.78 ± 0.16	<0.001
Postoperative BCVA, LogMAR	0.30 ± 0.14	0.20 ± 0.07	0.21 ± 0.08	0.44 ± 0.06	0.46 ± 0.07	<0.001
Visual acuity gain, LogMAR	0.38 ± 0.16	0.43 ± 0.16	0.46 ± 0.15	0.28 ± 0.13	0.32 ± 0.14	<0.001
UCLA Loneliness Scale score	41.6 ± 10.3	34.3 ± 4.9	52.9 ± 5.7	34.8 ± 4.3	52.7 ± 5.4	<0.001
Baseline MoCA score	27.7 ± 0.8	27.7 ± 0.8	27.6 ± 0.7	27.8 ± 0.8	27.6 ± 0.8	0.417
Barthel Index	84.5 ± 8.9	88.0 ± 8.0	86.1 ± 8.3	83.4 ± 8.0	76.8 ± 6.8	<0.001
Ocular comorbidity						0.010
No	341 (90.5%)	153 (94.4%)	67 (94.4%)	56 (82.4%)	65 (85.5%)	
Yes	36 (9.5%)	9 (5.6%)	4 (5.6%)	12 (17.6%)	11 (14.5%)	
Postoperative complication						0.721
No	358 (95.0%)	155 (95.7%)	67 (94.4%)	63 (92.6%)	73 (96.1%)	
Yes	19 (5.0%)	7 (4.3%)	4 (5.6%)	5 (7.4%)	3 (3.9%)	
Hypertension						0.722
No	246 (65.3%)	109 (67.3%)	45 (63.4%)	46 (67.6%)	46 (60.5%)	
Yes	131 (34.7%)	53 (32.7%)	26 (36.6%)	22 (32.4%)	30 (39.5%)	
Diabetes mellitus						0.002
No	297 (78.8%)	127 (78.4%)	66 (93.0%)	52 (76.5%)	52 (68.4%)	
Yes	80 (21.2%)	35 (21.6%)	5 (7.0%)	16 (23.5%)	24 (31.6%)	
History of stroke						0.243
No	326 (86.5%)	145 (89.5%)	60 (84.5%)	60 (88.2%)	61 (80.3%)	
Yes	51 (13.5%)	17 (10.5%)	11 (15.5%)	8 (11.8%)	15 (19.7%)	
Depression						0.006
No	353 (93.6%)	158 (97.5%)	67 (94.4%)	63 (92.6%)	65 (85.5%)	
Yes	24 (6.4%)	4 (2.5%)	4 (5.6%)	5 (7.4%)	11 (14.5%)	
Hearing impairment						0.039
No	316 (83.8%)	136 (84.0%)	60 (84.5%)	63 (92.6%)	57 (75.0%)	
Yes	61 (16.2%)	26 (16.0%)	11 (15.5%)	5 (7.4%)	19 (25.0%)	

Data are presented as mean ± standard deviation for continuous variables and n (%) for categorical variables. P values were calculated using Welch one-way analysis of variance for continuous variables and Fisher's exact test for categorical variables. G1, good visual recovery without loneliness; G2, good visual recovery with loneliness; G3, poor visual recovery without loneliness; G4, poor visual recovery with loneliness. BCVA, best-corrected visual acuity; LogMAR, logarithm of the minimum angle of resolution; UCLA, University of California Los Angeles; MoCA, Montreal Cognitive Assessment.

### 3.3 Incidence of cognitive impairment and univariate comparisons

At the 6-month follow-up, cognitive impairment was identified in 79 of 377 patients (21.0%). Its occurrence differed across the four joint exposure groups (*P* < 0.001): 4.9% in G1, 18.3% in G2, 7.4% in G3, and 69.7% in G4 (Figure 2). In the univariate comparisons, the cognitive impairment group included a higher proportion of patients with poor postoperative visual acuity status and baseline loneliness. These patients also had higher preoperative and postoperative LogMAR values, less visual acuity gain, higher UCLA Loneliness Scale scores, and lower baseline MoCA and Barthel Index scores. Ocular comorbidity and depression were more frequent in this group. No statistically significant differences were observed for age, sex, education duration, postoperative complications, hypertension, diabetes mellitus, history of stroke, or hearing impairment (Table 2).

**Figure 2:**
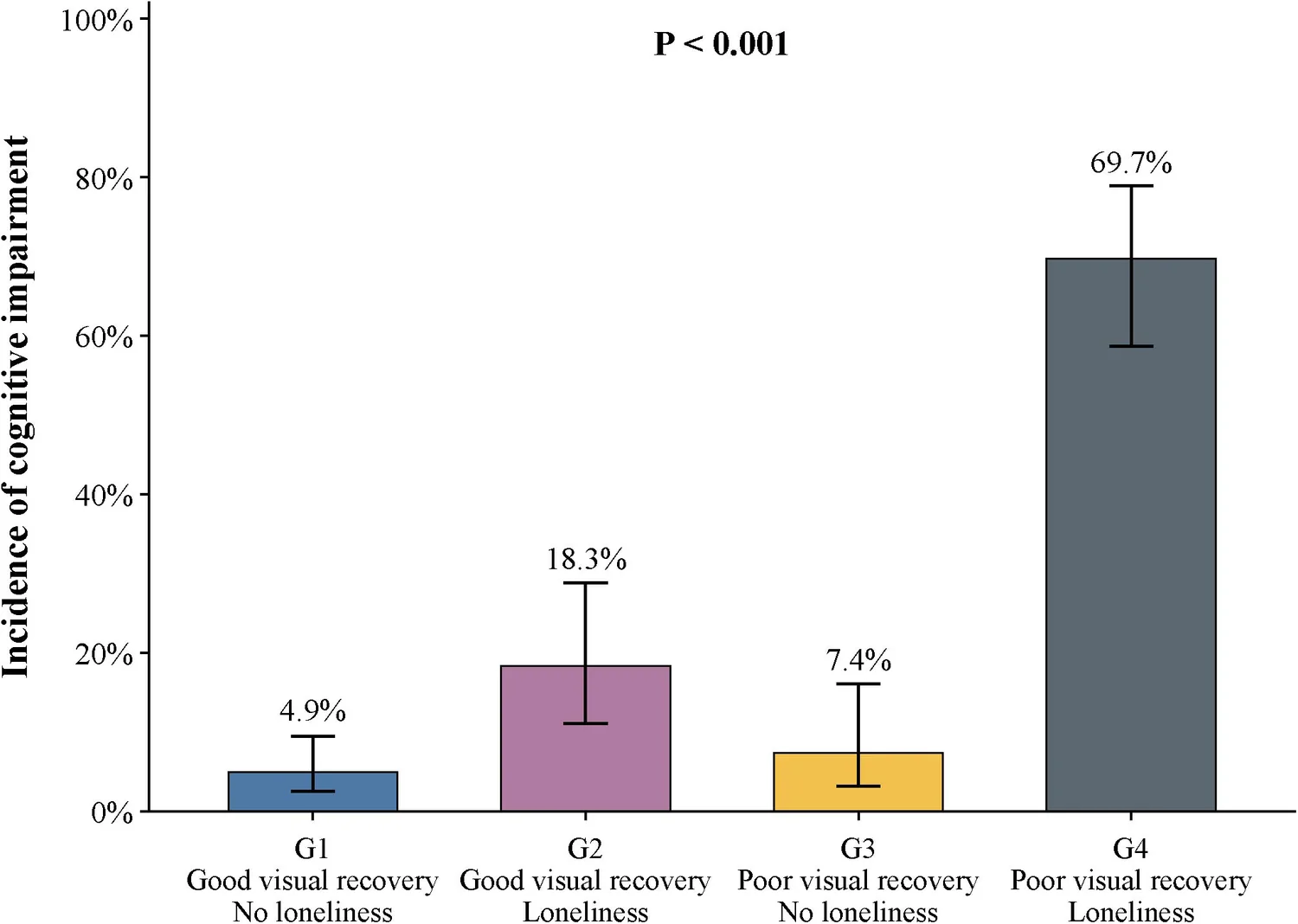
Incidence of cognitive impairment across the four joint exposure groups. The bars show the proportion of patients who developed cognitive impairment during follow-up in each group, and the error bars represent 95% confidence intervals calculated using the Wilson method. G1, good visual recovery without loneliness; G2, good visual recovery with loneliness; G3, poor visual recovery without loneliness; G4, poor visual recovery with loneliness. MoCA, Montreal Cognitive Assessment.

**Table 2:** Univariate comparison between patients with and without cognitive impairment.

Variable	Overall *N* = 377	No cognitive impairment *N* = 298	Cognitive impairment *N* = 79	*P* value
Joint exposure group				<0.001
G1: Good visual recovery without loneliness	162 (43.0%)	154 (51.7%)	8 (10.1%)	
G2: Good visual recovery with loneliness	71 (18.8%)	58 (19.5%)	13 (16.5%)	
G3: Poor visual recovery without loneliness	68 (18.0%)	63 (21.1%)	5 (6.3%)	
G4: Poor visual recovery with loneliness	76 (20.2%)	23 (7.7%)	53 (67.1%)	
Visual recovery status				<0.001
Good visual recovery	233 (61.8%)	212 (71.1%)	21 (26.6%)	
Poor visual recovery	144 (38.2%)	86 (28.9%)	58 (73.4%)	
Loneliness status				<0.001
No loneliness	230 (61.0%)	217 (72.8%)	13 (16.5%)	
Loneliness	147 (39.0%)	81 (27.2%)	66 (83.5%)	
Age, years	77.0 ± 7.1	76.7 ± 6.9	78.1 ± 7.9	0.151
Sex				0.515
Male	143 (37.9%)	116 (38.9%)	27 (34.2%)	
Female	234 (62.1%)	182 (61.1%)	52 (65.8%)	
Education duration, years	8.5 ± 3.9	8.6 ± 3.8	8.5 ± 4.3	0.845
Preoperative BCVA, LogMAR	0.68 ± 0.18	0.67 ± 0.18	0.72 ± 0.19	0.037
Postoperative BCVA, LogMAR	0.30 ± 0.14	0.27 ± 0.13	0.38 ± 0.14	<0.001
Visual acuity gain, LogMAR	0.38 ± 0.16	0.40 ± 0.16	0.34 ± 0.16	0.005
UCLA Loneliness Scale score	41.6 ± 10.3	39.5 ± 9.5	49.7 ± 9.0	<0.001
Baseline MoCA score	27.7 ± 0.8	27.8 ± 0.8	27.2 ± 0.8	<0.001
Barthel Index	84.5 ± 8.9	85.7 ± 8.7	80.2 ± 8.3	<0.001
Ocular comorbidity				0.029
No	341 (90.5%)	275 (92.3%)	66 (83.5%)	
Yes	36 (9.5%)	23 (7.7%)	13 (16.5%)	
Postoperative complication				0.386
No	358 (95.0%)	281 (94.3%)	77 (97.5%)	
Yes	19 (5.0%)	17 (5.7%)	2 (2.5%)	
Hypertension				0.086
No	246 (65.3%)	201 (67.4%)	45 (57.0%)	
Yes	131 (34.7%)	97 (32.6%)	34 (43.0%)	
Diabetes mellitus				0.353
No	297 (78.8%)	238 (79.9%)	59 (74.7%)	
Yes	80 (21.2%)	60 (20.1%)	20 (25.3%)	
History of stroke				0.063
No	326 (86.5%)	263 (88.3%)	63 (79.7%)	
Yes	51 (13.5%)	35 (11.7%)	16 (20.3%)	
Depression				0.017
No	353 (93.6%)	284 (95.3%)	69 (87.3%)	
Yes	24 (6.4%)	14 (4.7%)	10 (12.7%)	
Hearing impairment				0.492
No	316 (83.8%)	252 (84.6%)	64 (81.0%)	
Yes	61 (16.2%)	46 (15.4%)	15 (19.0%)	

Data are presented as mean ± standard deviation for continuous variables and n (%) for categorical variables. Percentages were calculated within each cognitive impairment group. P values were calculated using Welch's t-test for continuous variables and Fisher's exact test for categorical variables. Cognitive impairment was defined according to the prespecified MoCA cutoff at follow-up. G1, good visual recovery without loneliness; G2, good visual recovery with loneliness; G3, poor visual recovery without loneliness; G4, poor visual recovery with loneliness. BCVA, best-corrected visual acuity; LogMAR, logarithm of the minimum angle of resolution; UCLA, University of California Los Angeles; MoCA, Montreal Cognitive Assessment.

### 3.4 Multivariable association between joint exposure groups and cognitive impairment

Using G1 as the reference group, binary logistic regression showed that G2 was associated with higher odds of cognitive impairment in the crude model (OR, 4.31; 95% CI, 1.73–11.41; *P* = 0.002). The association remained after sequential adjustment and was similar in the fully adjusted model (OR, 5.39; 95% CI, 1.91–16.22; *P* = 0.002). For G3, the association was not statistically significant in either the crude or adjusted analyses. The fully adjusted OR was 1.85 (95% CI, 0.48–6.60; *P* = 0.346), although the wide confidence interval indicated limited precision. G4 showed the strongest association. The crude OR was 44.36 (95% CI, 19.67–112.22; *P* < 0.001), and the fully adjusted OR was 88.50 (95% CI, 27.43–338.63; *P* < 0.001). The forest plot showed the same overall pattern, with the confidence intervals for G2 and G4 remaining above 1, whereas that for G3 crossed 1 (Table 3 and Figure 3). Formal interaction analyses were then performed. A positive multiplicative interaction was observed between poor postoperative visual status and baseline loneliness (interaction OR, 8.85; 95% CI, 1.79–43.76; *P* = 0.006). Evidence of additive interaction was also present, with a relative excess risk due to interaction of 8.97 (95% CI, 2.72–15.22; *P* = 0.005), an attributable proportion of 0.69 (95% CI, 0.52–0.86; *P* < 0.001), and a synergy index of 3.88 (95% CI, 1.83–8.22; *P* < 0.001). These findings were consistent with a joint association that was greater than expected from the individual associations of postoperative visual status and baseline loneliness (Supplementary Table 1).

**Table 3:** Logistic regression analysis of the association between joint exposure groups and cognitive impairment.

	Crude model		Model 1		Model 2		Model 3	
Joint exposure group	OR (95% CI)	*P* value	OR (95% CI)	*P* value	OR (95% CI)	*P* value	OR (95% CI)	*P* value
G1: Good visual recovery without loneliness	Reference		Reference		Reference		Reference	
G2: Good visual recovery with loneliness	4.31 (1.73–11.41)	0.002	4.37 (1.74-11.57)	0.002	5.37 (2.02-15.14)	<0.001	5.39 (1.91-16.22)	0.002
G3: Poor visual recovery without loneliness	1.53 (0.45–4.76)	0.472	1.58 (0.46-4.92)	0.442	2.08 (0.58-6.91)	0.237	1.85 (0.48-6.60)	0.346
G4: Poor visual recovery with loneliness	44.36 (19.67–112.22)	<0.001	44.35 (19.60-112.58)	<0.001	90.16 (33.67-281.11)	<0.001	88.50 (27.43-338.63)	<0.001

Odds ratios were estimated using binary logistic regression, with G1 as the reference group. Crude model was unadjusted. Model 1 was adjusted for age, sex, and education duration. Model 2 was further adjusted for baseline MoCA score. Model 3 was further adjusted for Barthel Index, ocular comorbidity, postoperative complication, hypertension, diabetes mellitus, history of stroke, depression, and hearing impairment. G1, good visual recovery without loneliness; G2, good visual recovery with loneliness; G3, poor visual recovery without loneliness; G4, poor visual recovery with loneliness. OR, odds ratio; CI, confidence interval; MoCA, Montreal Cognitive Assessment.

**Figure 3:**
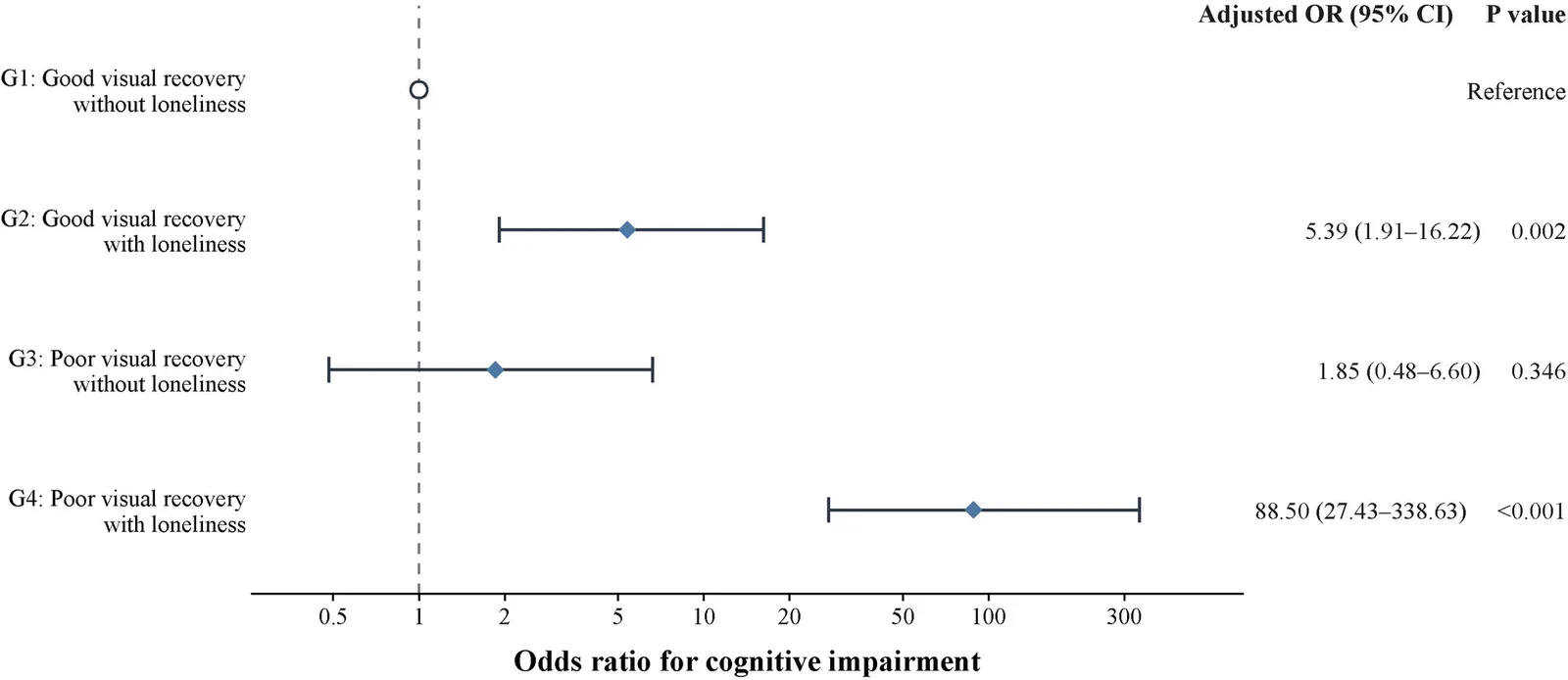
Forest plot of adjusted odds ratios for cognitive impairment across the four joint exposure groups. Odds ratios were estimated using binary logistic regression, with G1 as the reference group. The model was adjusted for age, sex, education duration, baseline MoCA score, Barthel Index, ocular comorbidity, postoperative complication, hypertension, diabetes mellitus, history of stroke, depression, and hearing impairment. G1, good visual recovery without loneliness; G2, good visual recovery with loneliness; G3, poor visual recovery without loneliness; G4, poor visual recovery with loneliness. OR, odds ratio; CI, confidence interval; MoCA, Montreal Cognitive Assessment.

### 3.5 Joint exposure risk estimates, cognitive change, and sensitivity analyses

The absolute proportion of patients with cognitive impairment differed markedly across the four joint exposure groups, ranging from 4.9% in G1 to 69.7% in G4 (Table 4). When cognitive change was analyzed as a continuous outcome, higher baseline UCLA Loneliness Scale scores were associated with a greater reduction in MoCA score over follow-up (r = −0.503, *P* < 0.001). The inverse pattern was present in both postoperative visual acuity strata and appeared steeper among patients with poor postoperative visual acuity status (Figure 4). MoCA change also differed across the four joint exposure groups (*P* < 0.001). G4 showed the largest decline, whereas G1 showed the smallest change (Supplementary Figure 1).

**Table 4:** Association between joint exposure groups and cognitive impairment.

			Crude model		Fully adjusted model	
Joint exposure group	Total *N*	Cognitive impairment, n/N (%)	OR (95% CI)	*P* value	OR (95% CI)	*P* value
G1: Good visual recovery without loneliness	162	8/162 (4.9%)	Reference		Reference	
G2: Good visual recovery with loneliness	71	13/71 (18.3%)	4.31 (1.73–11.41)	0.002	5.39 (1.91–16.22)	0.002
G3: Poor visual recovery without loneliness	68	5/68 (7.4%)	1.53 (0.45–4.76)	0.472	1.85 (0.48–6.60)	0.346
G4: Poor visual recovery with loneliness	76	53/76 (69.7%)	44.36 (19.67–112.22)	<0.001	88.50 (27.43–338.63)	<0.001

Data are presented as events/total cases with incidence percentages. Odds ratios were estimated by binary logistic regression, using G1 as the reference group. The crude model was unadjusted, whereas the fully adjusted model included age, sex, education duration, baseline MoCA score, Barthel Index, ocular comorbidity, postoperative complication, hypertension, diabetes mellitus, history of stroke, depression, and hearing impairment. G1, good visual recovery without loneliness; G2, good visual recovery with loneliness; G3, poor visual recovery without loneliness; G4, poor visual recovery with loneliness. OR, odds ratio; CI, confidence interval; MoCA, Montreal Cognitive Assessment.

**Figure 4:**
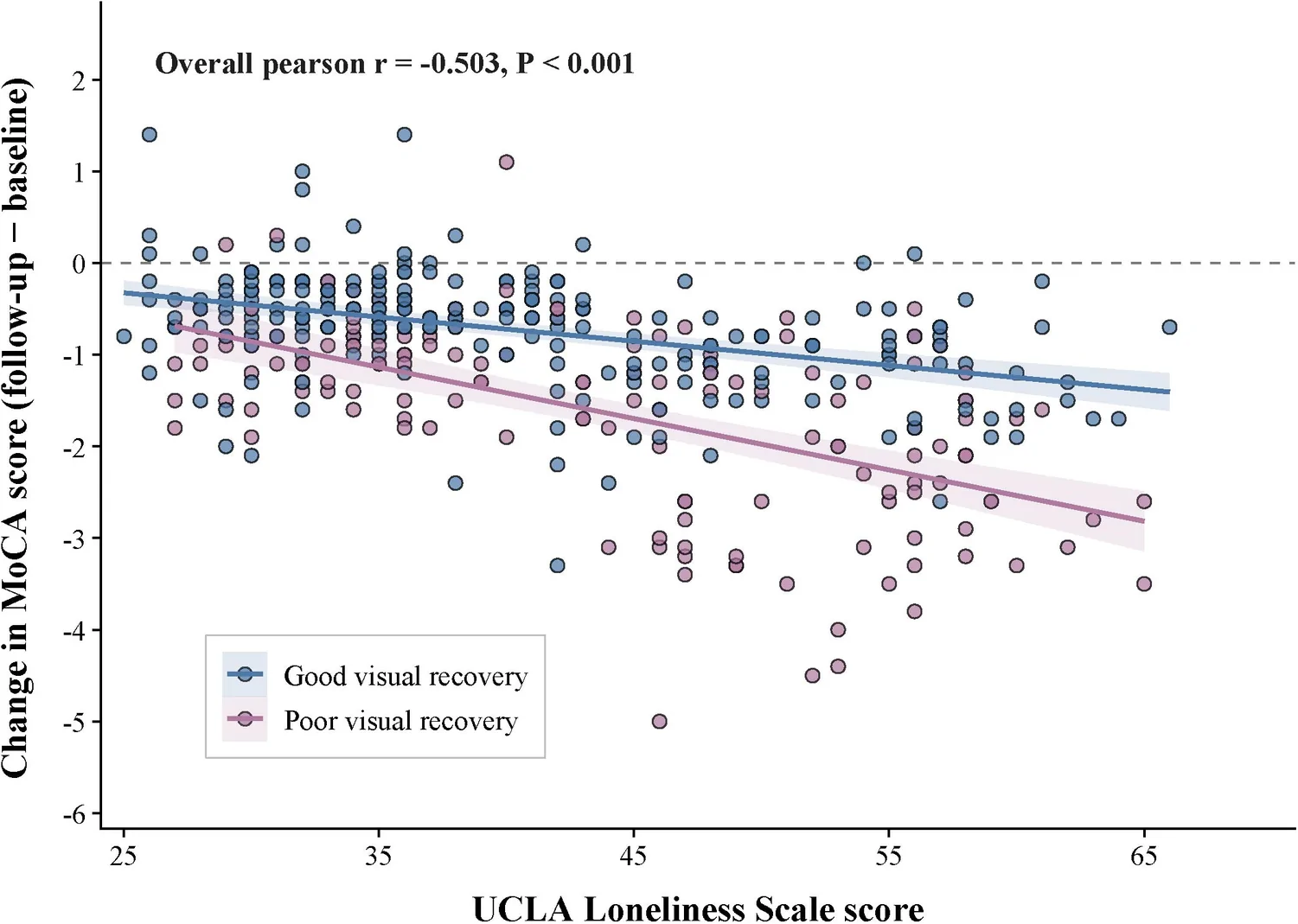
Relationship between loneliness and cognitive change. Change in MoCA score was defined as the follow-up score minus the baseline score; more negative values indicate greater cognitive decline. Points represent individual patients. Fitted lines and shaded bands show linear trends with 95% confidence intervals according to visual recovery status. The overall Pearson correlation coefficient is displayed in the figure. UCLA, University of California Los Angeles; MoCA, Montreal Cognitive Assessment.

The direction of the associations was generally similar across the alternative covariate-adjustment models. G2 remained associated with higher odds of cognitive impairment, with adjusted ORs ranging from 5.32 to 5.39. The estimates for G3 did not reach statistical significance, although their confidence intervals were wide and did not exclude a potentially meaningful association. G4 consistently had the largest effect estimate, with adjusted ORs ranging from 88.50 to 100.23 (all *P* < 0.001) (Supplementary Table 2). Firth penalized logistic regression produced smaller estimates while preserving the direction of the associations for G2 and G4. Compared with G1, the adjusted OR was 4.75 for G2 (95% CI, 1.77–13.50; *P* = 0.002), 1.84 for G3 (95% CI, 0.52–6.08; *P* = 0.332), and 62.18 for G4 (95% CI, 20.67–218.82; *P* < 0.001) (Supplementary Table 3). These findings suggest that the overall pattern was not entirely dependent on conventional maximum-likelihood estimation. Nevertheless, the small and uneven numbers of events across the joint exposure groups, together with the wide confidence interval for G4, indicate substantial uncertainty in the precise magnitude of the estimates. Sparse data and model specification may therefore still have influenced the results.

## 4 Discussion

Visual impairment and social disconnection are increasingly recognized as factors relevant to cognition in later life (22–24). Recent evidence suggests that cataract surgery may be associated with better cognitive outcomes; however, most studies have focused on whether surgery was performed rather than on the level of visual acuity achieved afterward (25–27). In the present study, the occurrence of cognitive impairment at 6 months differed markedly across the four joint exposure groups. It was lowest among patients with good postoperative visual acuity status and no baseline loneliness and highest among those with both poor postoperative visual acuity status and baseline loneliness. These findings suggest that considering postoperative visual acuity and loneliness together may provide a more complete description of postoperative cognitive vulnerability than considering either factor alone.

The associations differed across the joint exposure groups. Poor postoperative visual acuity without baseline loneliness was not significantly associated with cognitive impairment after adjustment, although the wide confidence interval indicates limited precision and does not rule out a potentially meaningful association. Baseline loneliness remained associated with higher odds of cognitive impairment among patients with good postoperative visual acuity, while the largest estimate was observed in those with both poor postoperative visual acuity and baseline loneliness. This large estimate partly reflects the marked difference in cognitive impairment between the dual-exposure group and the reference group, but its magnitude should not be viewed as precise because some groups contained few outcome events. Interaction analyses suggested departures from both multiplicative and additive models. These findings should be regarded as exploratory and do not establish a causal or biological synergistic effect. The direction of the associations was broadly similar across the crude, adjusted, and Firth penalized models, lending support to the overall pattern while leaving uncertainty about the exact size of the estimates.

The findings concerning loneliness are consistent with previous evidence linking loneliness and reduced social connection to poorer cognitive outcomes in older adults (28–31). In our cohort, higher UCLA scores were associated with a more negative change in MoCA scores. The fitted relationship appeared steeper among patients with poor postoperative visual acuity, and the dual-exposure group showed the greatest decline in cognitive scores. Persistent visual difficulty may restrict daily activities and social participation, while loneliness may further reduce opportunities for cognitive stimulation and emotional support. Depressive symptoms, disturbed sleep, stress-related responses, inflammation, and reduced social engagement have been proposed as possible pathways (32). These mechanisms were not measured in the present study and should therefore be viewed as possible explanations rather than established causal pathways.

These findings may have practical relevance for the follow-up of older adults after cataract surgery. Routine postoperative care appropriately focuses on visual acuity, refractive outcomes, and the detection of ocular complications (33). For older patients, however, postoperative wellbeing may also depend on functional capacity, emotional health, social participation, and the ability to resume everyday activities. Vision loss and social disconnection have both been identified as potentially modifiable factors associated with cognitive health in later life (34). Assessment of baseline loneliness alongside postoperative best-corrected visual acuity may therefore provide additional information when clinicians consider which patients warrant closer cognitive or functional follow-up. Poor postoperative visual acuity may continue to limit mobility, confidence, and social engagement, while loneliness may indicate fewer opportunities for stimulation or support. Loneliness screening should not replace cognitive assessment, and the present findings do not establish that loneliness causes cognitive impairment. Rather, loneliness may serve as an accessible marker of broader vulnerability. Longitudinal and meta-analytic studies have linked loneliness and social isolation with cognitive decline, mild cognitive impairment, and dementia-related outcomes (34–36), while studies of cataract surgery suggest that improved vision may be associated with better cognitive outcomes in older adults (26, 34). Older adults with both poor postoperative visual acuity and loneliness may therefore warrant closer attention to cognition, daily functioning, mood, and social support. A person-centered approach that considers broader intrinsic capacity, rather than focusing exclusively on ocular outcomes, is consistent with contemporary geriatric care models (37).

Several limitations should be considered when interpreting these findings. The retrospective, single-center design precludes causal inference and may have introduced selection bias. We used a complete-case approach and excluded patients with cognitive impairment at baseline so that the 6-month outcome would represent newly identified impairment. This decision improved the clarity of the outcome definition, but it also produced a cognitively healthier study population and may limit the applicability of the findings to patients with pre-existing cognitive deficits. Residual confounding is another concern. Depression was included in the adjusted models, although the medical records did not consistently capture symptom severity or changes over time. Information on living arrangement, social participation, physical activity, sleep, frailty, and socioeconomic circumstances was also incomplete, despite the possibility that these factors are related to both loneliness and cognition (38–41). Some misclassification may have arisen from the operational thresholds used to define postoperative visual acuity status and baseline loneliness. The available records also did not allow poor postoperative visual acuity to be attributed reliably to a specific ocular condition or surgical complication. Cognitive function was measured before surgery and again at 6 months, whereas loneliness was assessed only at baseline. We could therefore examine cognitive change according to postoperative BCVA, but not whether loneliness itself changed after surgery or whether such change differed by visual outcome. Repeated assessment of both constructs would be useful because loneliness and cognition may evolve together over time (42). The MoCA is a screening tool rather than a diagnostic assessment, and use of the same version at both visits may have introduced a practice effect that partly obscured cognitive decline (43). Blinding of assessors to exposure status could not be verified from the routine records. The event distribution across the joint exposure groups was also uneven. Only 8 cognitive impairment events occurred in G1 and 5 in G3, while the fully adjusted model included several covariates. This imbalance may have contributed to sparse-data bias, unstable coefficients, and wide confidence intervals. Firth penalized logistic regression reduced the estimates and preserved their direction, but it could not remove the uncertainty arising from the limited sample size or model specification. The interaction analyses were subject to the same limitation and should be regarded as exploratory rather than as evidence of a definitive synergistic effect. Larger prospective, multicenter studies should include patients with a broader range of baseline cognitive function, collect more detailed ophthalmic and social data, and repeat assessments of visual acuity, loneliness, and cognition. Alternate MoCA forms or a fuller neuropsychological battery may also provide a more reliable estimate of postoperative cognitive change.

## 5 Conclusion

Among older adults undergoing cataract surgery, baseline loneliness was associated with higher odds of cognitive impairment at 6 months, with the largest estimate observed in patients who also had poor postoperative best-corrected visual acuity. Poor postoperative visual acuity in the absence of loneliness was not significantly associated with cognitive impairment, although the wide confidence interval precludes a firm conclusion. The direction of the findings was similar across conventional and Firth penalized regression models, but the large effect estimates and wide confidence intervals indicate limited precision. Exploratory analyses suggested interaction on both multiplicative and additive scales; these findings should not be interpreted as evidence of a definitive synergistic or causal effect. Assessment of postoperative visual acuity together with baseline loneliness may help identify patients who warrant closer cognitive, functional, and psychosocial follow-up. Larger prospective, multicenter studies are needed to confirm these associations and provide more precise estimates.

## Data Availability

The raw data supporting the conclusions of this article will be made available by the authors, without undue reservation.
