# Supplementary material for: Joint association of postoperative visual acuity status and baseline loneliness with cognitive impairment after cataract surgery in older adults: a retrospective cohort study
Source: Front Med (Lausanne). 2026 Sep 15;13:1948093. doi: 10.3389/fmed.2026.1948093 (PMC13619495; doi:10.3389/fmed.2026.1948093)
Supplement: Supplementary file 1 [file Datasheet1.docx]

Supplementary Material

# 1 Supplementary Figures and Tables

## 1.1 Supplementary Figures

**Supplementary Figure 1.** **Distribution of change in MoCA score across the four joint exposure groups.**

Change in MoCA score was calculated as the follow-up MoCA score minus the baseline MoCA score, with lower values indicating greater cognitive decline. Violin plots show the distribution of MoCA change, box plots show the median and interquartile range, and points represent individual patients. The P value represents the overall comparison among the four groups and was calculated using Welch one-way analysis of variance. G1, good visual recovery without loneliness; G2, good visual recovery with loneliness; G3, poor visual recovery without loneliness; G4, poor visual recovery with loneliness. MoCA, Montreal Cognitive Assessment.

## 1.2 Supplementary Tables

**Supplementary Table 1. Interaction between postoperative visual status and baseline loneliness for 6-month cognitive impairment**

| **Analysis or contrast** | **Cognitive impairment, n/N (%)** | **Effect measure** | **Adjusted estimate (95% CI)** | ***P* value** |
| --- | --- | --- | --- | --- |
| **Joint exposure categories** | | | | |
| G1: Good postoperative visual status + no baseline loneliness | 8/162 (4.9%) | OR | 1.00 (Reference) | — |
| G2: Good postoperative visual status + baseline loneliness | 13/71 (18.3%) | OR | 5.39 (1.91–16.22) | 0.002 |
| G3: Poor postoperative visual status + no baseline loneliness | 5/68 (7.4%) | OR | 1.85 (0.48–6.60) | 0.346 |
| G4: Poor postoperative visual status + baseline loneliness | 53/76 (69.7%) | OR | 88.50 (27.43–338.63) | <0.001 |
| **Multiplicative interaction** | | | | |
| Poor postoperative visual status × baseline loneliness |  | Interaction OR | 8.85 (1.79–43.76) | 0.006 |
| **Additive interaction** | | | | |
| Relative excess risk due to interaction |  | RERI | 8.97 (2.72–15.22) | 0.005 |
| Attributable proportion due to interaction |  | AP | 0.69 (0.52–0.86) | <0.001 |
| Synergy index |  | SI | 3.88 (1.83–8.22) | <0.001 |

Values are presented as events/total (%) or adjusted estimates with 95% confidence intervals. ORs for the joint exposure categories were estimated using multivariable binary logistic regression, with G1 as the reference group. The multiplicative interaction estimate is reported as the interaction OR; its 95% CI was calculated using the Wald method, whereas the P value was obtained from a one-degree-of-freedom likelihood-ratio test comparing models with and without the interaction term. Additive interaction was evaluated on the adjusted risk-ratio scale using modified Poisson regression with an HC0 robust sandwich covariance matrix, and 95% CIs for RERI, AP, and SI were calculated using the delta method. The adjusted RRs for G2, G3, and G4 versus G1 were 3.55, 1.56, and 13.09, respectively. All models were adjusted for age, sex, education duration, baseline MoCA score, Barthel Index, ocular comorbidity, postoperative complication, hypertension, diabetes mellitus, history of stroke, depression, and hearing impairment. The null values are 0 for RERI and AP and 1 for SI. OR, odds ratio; RR, risk ratio; CI, confidence interval; RERI, relative excess risk due to interaction; AP, attributable proportion due to interaction; SI, synergy index; LogMAR, logarithm of the minimum angle of resolution; UCLA, University of California, Los Angeles; MoCA, Montreal Cognitive Assessment.

**Supplementary Table 2. Sensitivity analyses of the association between joint exposure groups and cognitive impairment**

|  | **Model A** | | **Model B** | | **Model C** | |
| --- | --- | --- | --- | --- | --- | --- |
| **Joint exposure group** | **OR (95% CI)** | **P value** | **OR (95% CI)** | **P value** | **OR (95% CI)** | **P value** |
| **G1: Good visual recovery without loneliness** | Reference |  | Reference |  | Reference |  |
| G2: Good visual recovery with loneliness | 5.39 (1.91–16.22) | 0.002 | 5.32 (1.97–15.26) | 0.001 | 5.37 (2.02–15.14) | <0.001 |
| G3: Poor visual recovery without loneliness | 1.85 (0.48–6.60) | 0.346 | 2.24 (0.60–7.76) | 0.206 | 2.08 (0.58–6.91) | 0.237 |
| G4: Poor visual recovery with loneliness | 88.50 (27.43–338.63) | <0.001 | 100.23 (31.60–376.98) | <0.001 | 90.16 (33.67–281.11) | <0.001 |

Odds ratios were estimated using binary logistic regression, with G1 as the reference group. Model A was adjusted for age, sex, education duration, baseline MoCA score, Barthel Index, ocular comorbidity, postoperative complication, hypertension, diabetes mellitus, history of stroke, depression, and hearing impairment. Model B was adjusted for age, sex, education duration, baseline MoCA score, Barthel Index, history of stroke, and depression. Model C was adjusted for age, sex, education duration, and baseline MoCA score. G1, good visual recovery without loneliness; G2, good visual recovery with loneliness; G3, poor visual recovery without loneliness; G4, poor visual recovery with loneliness. OR, odds ratio; CI, confidence interval; MoCA, Montreal Cognitive Assessment.

**Supplementary Table 3. Sensitivity analysis of joint exposure groups and 6-month cognitive impairment using Firth penalized logistic regression**

| **Joint exposure group** | **Cognitive impairment, n/N (%)** | **Conventional multivariable logistic regression** | | **Firth penalized logistic regression** | |
| --- | --- | --- | --- | --- | --- |
|  |  | **Adjusted OR (95% CI)** | ***P* value** | **Adjusted OR (95% CI)** | ***P* value** |
| G1: Good visual status, no baseline loneliness | 8/162 (4.9%) | Reference | — | Reference | — |
| G2: Good visual status, baseline loneliness | 13/71 (18.3%) | 5.39 (1.91–16.22) | 0.002 | 4.75 (1.77–13.50) | 0.002 |
| G3: Poor visual status, no baseline loneliness | 5/68 (7.4%) | 1.85 (0.48–6.60) | 0.346 | 1.84 (0.52–6.08) | 0.332 |
| G4: Poor visual status, baseline loneliness | 53/76 (69.7%) | 88.50 (27.43–338.63) | <0.001 | 62.18 (20.67–218.82) | <0.001 |

Data are presented as events/total (%) or adjusted odds ratios (ORs) with 95% confidence intervals (CIs). Both models were adjusted for age, sex, education duration, baseline MoCA score, Barthel Index, ocular comorbidity, postoperative complication, hypertension, diabetes mellitus, history of stroke, depression, and hearing impairment. Firth estimates, 95% CIs, and P values were derived from profile penalized likelihood; G1 was the reference group. LogMAR, logarithm of the minimum angle of resolution; UCLA, University of California, Los Angeles; MoCA, Montreal Cognitive Assessment; OR, odds ratio; CI, confidence interval.
